# Various parts of *Carica* *papaya* L. - the cardioprotective potential and prospects for pharmacological use

**DOI:** 10.3389/fmolb.2026.1818282

**Published:** 2026-06-24

**Authors:** Beata Olas

**Affiliations:** Department of General Biochemistry, Faculty of Biology and Environmental Protection, University of Lodz, Lodz, Poland

**Keywords:** antioxidant, *C. papaya*, cardioprotective activity, papain, papaya

## Abstract

This mini-review describes the role of various parts of *C. papaya* L. (a member of the *Caricaceae* family) in the prevention and treatment of cardiovascular diseases (CVDs). *C. papaya* is mainly cultivated for its edible fruits, which are eaten fresh or made into candies, jams, drinks, or dried fruit. Not only fruits, but also other parts of this plant, including flowers, bark, and roots, have been traditionally used worldwide for the preparation of different medicinal preparations. For example, in traditional medicine, the fruit of *C. papaya* is used to normalize blood pressure due to its possible diuretic activity. The present mini-review paper also describes its cardioprotective potential in other details (not only anti-hypertensive activity). Moreover, cardioprotective mechanisms of the main bioactive compounds isolated from various parts of papaya were presented to provide a basis for further study and potential pharmacological use.

## 1 Introduction

About 20,000 plant species, including exotic plants (for example, *Carica papaya* L., which is a member of the *Caricaceae* family, known as “papaya,” “papaia,” “pawpaw” or “kates”) are used in different traditional medicines, and they are considered potential reservoirs for the discovery of new drugs and supplements (Chaachouay and Zidane, 2024; Sindhu et al., 2019; Mariano et al., 2022).

According to the Food and Agriculture Organization of the United Nations (FAO), about 7 million tons of papaya fruits are produced worldwide, especially in West India, China, and Central and South America (FAO, 2025).

*C. papaya* is a tropical, evergreen, woody-like herb that usually grows as a single, unbranched stem, reaching heights of 2–10 m. *C. papaya* is mainly cultivated for its edible fruits, which are large, fleshy berries, often oval to pear-shaped, with a yellow-green skin and orange-yellow flesh, weighing from 0.5 kg to several kilograms. Fruits are eaten fresh or made into candies, jams, drinks, or dried fruit. It can also be eaten raw in salads, pickled, added to muesli, and processed into smoothies or wine, at both unripe and ripe stages. Green fruits of papaya are also used in cooking (Sindhu et al., 2019; Vij and Prashar, 2015; Singh et al., 2020). Green fruits of *C. papaya* are used in preparations without carotene, for example, juices, ice creams, cakes, and salads (Oloyede, 2005; Parle and Gurditta, 2011; Krishna et al., 2008). Moreover, latex from green fruits is used in beverage, food, and pharmaceutical industries for the production of chewing gum, extraction of fish oil, degumming natural silk, chill-proofing beer, tenderizing meat, and treating digestive disorders (Sindhu et al., 2019). According to Alara et al. (2020), papaya fruit has been ranked among the top five fruits together with grapefruit, kiwi, watermelon, and guava on the basis of its nutritional score.

Papaya is also cultivated for other parts of the plant, such as bark, flowers, and roots, which have been traditionally used worldwide for the preparation of different medicinal preparations (Sindhu et al., 2019; Vij and Prashar, 2015; Singh et al., 2020). However, roots and flowers have not cardioprotective potential. For example, medicinal use of flowers include treatment of cough, bronchitis, laryngitis and tracheitis in Asia. Roots are used in diseases of digestive system. In addition, fresh young leaves of papaya are consumed in various countries as leafy vegetables after steaming. For example, in some parts of Asia, they are eaten like spinach (Sindhu et al., 2019; Vij and Prashar, 2015; Sharma et al., 2020; Sharma et al., 2022; Kumar et al., 2020). The black seeds of papaya are also edible and have a spicy, sharp taste. They can be ground and used as a substitute for black pepper (Sindhu et al., 2019). Moreover, seeds and leaves of *C. papaya* are added to various food products, including flour and teas (Nwofia et al., 2012). Finally, papaya flowers are eaten in Java (Sindhu et al., 2019; Arvind et al., 2013).

Papaya fruit contains considerable amounts of various active compounds with cardioprotective potential that protect against hypercholesterolemia, oxidative stress, inflammatory process, and obesity (Vij and Prashar, 2015; Santana et al., 2019; Akanda et al., 2022; Arshad et al., 2025). Not only ripe and green fruits of papaya, but also its seeds and leaves contain different bioactive compounds. For example, the leaves of papaya have a high concentration of phenolic compounds and fibers. Their consumption can bring significant benefits to the cardiovascular system (Sharma et al., 2020; Sharma et al., 2022; Santana et al., 2019). They are also used in the treatment of various other diseases in traditional medicine. For example, dry papaya leaves are used as cigars for smoking by people suffering from respiratory diseases. Moreover, a decoction from fresh papaya leaves is added to tea to cure malaria (Sindhu et al., 2019; Vij and Prashar, 2015; Singh et al., 2020; Sharma et al., 2020; Kumar et al., 2020). Recently, researchers have also been focusing on health-promoting components present in *C. papaya* leaves and leaf powder. There are reports that papaya leaf extract can be used in the production of silver nanoparticles (Kumar et al., 2020; Banala et al., 2015; Dulta et al., 2018).

Although several review articles (Sindhu et al., 2019; Singh et al., 2020; Sharma et al., 2020; Santana et al., 2019) have described the phytochemical characteristics of various parts of papaya and suggested that this plant has medicinal value, these papers generally did not include information about its cardioprotective mechanisms. For example, Mariano et al. (2022) have demonstrated that in traditional medicine, the fruit of *C. papaya* is used to normalize blood pressure due to its possible diuretic activity. Therefore, for the first time, this mini-review paper describes the role of various parts of *C. papaya* in the prevention and treatment of cardiovascular diseases (CVDs), which is the leading cause of mortality worldwide, accounting for 32% of deaths in 2023. The present mini-review paper also describes its cardioprotective potential in other details (not only anti-hypertensive activity). Moreover, cardioprotective mechanisms of the main bioactive compounds isolated from various parts of papaya were presented to provide a basis for further study and potential pharmacological use.

## 2 Methodology for literature search

Both original and review papers were collected from various databases, including Web of Knowledge, PubMed, Science Direct, Google Scholar, Scopus, and Medline. For the search, various combinations of the keywords: “cardioprotective activity,” “cardiovascular disease,” “papaya,” “papaia,” “*C. papaya*,” and “*C. papaya*” were used. The last search was run on 2 December 2025. No time criteria were applied to the search, but recent papers were read first. Papers were first selected based on their relevance to the title of the present manuscript, and the identified articles were screened by reading the abstract. Any relevant identified papers were summarized. After obtaining the full texts of the included studies, the reference sections were also manually examined to identify any additional new papers.

## 3 Phytochemical composition of various parts of *C. papaya*

### 3.1 Fruits

Papaya fruits have a favorable cost-to-nutritional value ratio. Moreover, they have low caloric content (32 kcal/100 g ripe fruit) and are a good source of minerals and vitamins. Among the most commercialized fruits (apple, banana, orange, and watermelon), papaya has the highest concentration of fiber (0.8 g/100 g), which lowers cholesterol levels. Papaya fruits are also a good source of carotene (888 IU per 100 g of ripe fruit pulp) (Sindhu et al., 2019; Santana et al., 2019).

Studies report that papaya fruits are rich in many different bioactive compounds, including phenolics (flavonoids, terpenols, alkaloids, and saponins, among others) (Oloyede, 2005; Akanda et al., 2022). Bouanga-Kalou et al. (2011) detected considerable quantities of total phenolic compounds (203 mg/100 g extract) in the methanolic extract from *C. papaya* pulp. Recently, Jeon et al. (2022) have noted that total phenolic content differs between papaya extracts from unripe (235 ± 0.3 mg gallic acid equivalent (GAE)/100 g DW) and ripe peel-pulp (568 ± 0.3 mg GAE/100 g DW). They have identified three phenolic acids (cynarine, chlorogenic acid, and neochlorogenic acid) and two flavonoids (vicenin II and eupatorine) in the tested papaya extracts.

Green papaya fruits and leaves contain papain (EC. 3.4.22.2), a cysteine protease with action similar to that of pepsin in gastric juice. In addition, the latex from *C. pa-paya* is a complex mixture of chemical compounds which contains other proteinases (papaya protease IV (EC. 3.4.22.6), caricain (EC. 3.4.22.30), and chymopapain (EC. 3.4.22.25)), as well as kaempferol, quercetin, and p-coumaric acid (Singh et al., 2020). It is harvested from unripe fruits by making an incision on the fruit surface during a 4–5 day period and collecting the latex until it stops flowing (Sindhu et al., 2019).

Interestingly, papain can tenderize meat. Because of this property, papaya is added to marinades and sauces in certain cuisines (Sindhu et al., 2019; Santana et al., 2019). Papain (together with bromelain, a mixture of different proteolytic enzymes), is also an ingredient of various dietary supplements intended for people with digestive problems or people with low physical activity (Singh et al., 2020; Gracz-Bernaciak et al., 2021; Yang et al., 2023; Sethia et al., 2025). Recently, Fei et al. (2023) have observed that papain has anti-atherosclerotic properties as well.

Other studies report that papaya fruit juice is a source of lipids and fatty acids, including palmitic (16.2%), linoleic (6.15%), and stearic acids (4,7%) (Arvind et al., 2013).

Additionally, various phenolic compounds and carotenoids were detected in pa-paya peel. For example, Salla et al. (2016) reported that the extract from papaya peel contains significant amounts of ferulic acid (94.5 μg/mL), p-coumaric acid (38.2 μg/mL), caffeic acid (29.3 μg/mL), gallic acid (18.1 μg/mL), and quercetin (3.2 μg/mL).

### 3.2 Seeds

*C. papaya* seeds are also a source of various bioactive compounds, including phenolic compounds (total phenolic content – 957.6 mg/kg), tocopherols (α (51.8 mg/kg) and δ (18.9 mg/kg)), carotenoids (including β-carotene (65.6 IU per 100 g of seeds), glucosinolates, and isothiocyanate (Rani et al., 2023). Moreover, the oil from *C. papaya* seeds contains different fatty acids (stearic (4.7%), palmitic (16.2%), oleic (71.0%), and linoleic (6.1%) acids) and β-sitosterol (Sindhu et al., 2019; Santana et al., 2019; Miean and Mohamed, 2011; Neu et al., 2009; Doan et al., 2020). Doan et al. (2020) observed that papaya seed oil contains phenolic compounds (total phenolic content was 33.99 mg GAE/g). In addition, it showed antioxidant potential, which was measured by the 2,2-diphenyl-1-picrylhydrazyl (DPPH) method. Jeon et al. (2022) noted that the content of total phenolic compounds in the seeds decreases from 2070 to 1080 mg GAE/100 g DW during ripening.

### 3.3 Leaves

Papaya leaves contain carbohydrates, lipids, and proteins and therefore can be used as a nutritional agent. Some studies also indicate that *C. papaya* leaves contain a high content of phenolic compounds (especially flavonoids and pro-anthocyanins) and food fiber (13.1 g/100 g of leaves) (Sharma et al., 2020; Sharma et al., 2022; Abdel-Hsalim et al., 2020; Nandini et al., 2020). Sharma et al. (Sharma et al., 2020) identified caffeic acid, quercetin, protocatechuic acid, 5,7-dimethy coumarin, chlorogenic acid, and p-coumaric acid in the leaves. In addition, the presence of saponins, benzyl-isothiocyanate, tocopherol, various vitamins, and minerals was described (Singh et al., 2020). Moreover, the concentrations of calcium, potassium, iron, and magnesium are higher in papaya leaves than in fruits and seeds (for potassium: 257 mg/100 g of fruit pulp, 344 mg/100 g of seeds, and 534 mg/100 g of leaves) (Santana et al., 2019; Voung et al., 2013). Results of Bamisaye et al. (Bamisaye et al., 2013) demonstrated that aqueous extract from papaya leaves contains 0.001% tannins, 0.004% steroids, 0.011% phenolic compounds, 0.013% flavonoids, 0.019% alkaloids, and 0.022% saponins.

Recently, Chaijan et al. (2024) have examined the chemical composition of leaf extracts from three different cultivars of papaya. The dark green leaves of Holland, Thai Local, and Khaek Dam cultivars were used in the experiment. Their carbohydrate content ranged from 5.8% to 17.9%, the fat content ranged from 7.3% to 11.7%, the protein content ranged from 26.0% to 32.2%, and the fiber content ranged from 23.2% to 38.5%. The Thai Local cultivar possessed significantly higher carbohydrate and protein concentrations than the other two cultivars. Authors also identified various phenolic compounds (especially isorhamnetin, kaempferol, quercetin, sinapic acid, vanillin, ferulic acid, salicylic acid, isoferulic acid, and syringic acid; the total phenolic compounds content was 113.9–173.7 mg GAE/g extract), as well as terpenoids, indole, and glycosides.

**FIGURE 1:**
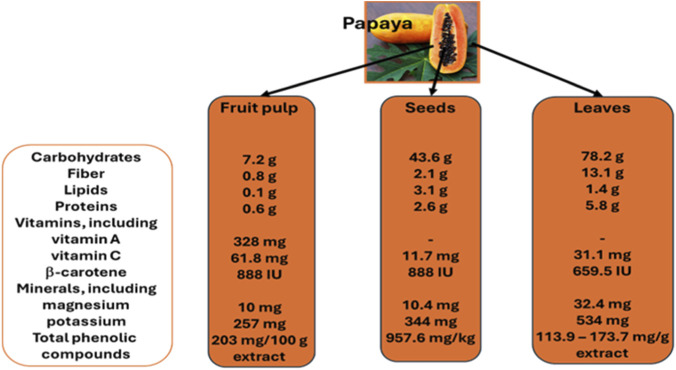
The content of macronutrients, fiber, total phenolic compounds, and selected vitamins and minerals of various parts of papaya (per 100 g of ripe fruit pulp, seeds, and leaves). Compilation of data (Santana et al., 2019; Miean and Mohamed, 2011; Neu et al., 2009; Voung et al., 2013).

The contents of macronutrients, fiber, total phenolic compounds, and selected vitamins and minerals in various parts of papaya (per 100 g of ripe fruit pulp, seeds, and leaves) are demonstrated in Figure 1. More details about the phytochemical characteristics of *C. papaya*, the identification/quantification methods, and the extraction methods are described in other review papers (Sindhu et al., 2019; Sharma et al., 2022; Santana et al., 2019).

## 4 Cardioprotective properties of extracts from various parts of papaya and it’s bioactive compounds

Preparations (especially extracts) from papaya have high levels of various bioactive compounds (for example, phenolic compounds, fiber, minerals, and vitamins) with different biological properties (including cardioprotective potential) (Akanda et al., 2022; Vega-Galvez et al., 2019), but strong scientific evidence of their efficacy is still lacking. Potential mechanisms of the cardioprotective effect of various compounds present in papaya include anti-obesity properties, anti-hypertensive activity, hypolipidemic potential, and anti-inflammatory action, among others. However, many studies that focus on this topic are limited to *in vitro* and animal models, and show considerable heterogeneity, making it difficult to compare results. Moreover, authors often do not describe the phytochemical profile of the studied extracts. For example, research showed that extracts from papaya leaves and seeds had hypolipidemic activity in animal subjects with poor health, including diabetic rats. However, these conclusions are based on studies conducted with a wide range of extracts from various parts of *C. papaya*, with different doses and experimental models, which contribute to large methodological heterogeneity. For example, extracts from leaves were supplemented between 120 and 500 mg/kg/bw/day (21–30 days), but there is no information about their cardioprotective potential during longer supplementation. In addition, dietary background, lifestyle variables, or genetic variability are rarely described by authors. This heterogeneity limits comparability across studies and affects the strength of conclusions, but this review sheds new light on the cardioprotective potential of papaya, which is summarized in Table 1. However, it is very difficult to propose whether tested doses (in animal models) may be physiologically relevant for humans.

**TABLE 1:** Cardioprotective action of extracts from different parts of *Carica papaya* and their components in various models.

Part of *C. papaya*/bioactive compound	Type of extract/dose	Experimental model	Main effects	Reference
Fruit extract	Crude extract/20 mg/kg bw	Wistar rats with hypertension (N = 15, *in vivo*)	Anti-hypertensive activity	Eno et al. (2000)
Seed extract	Aqueous extract/100–400 mg/kg bw (30 days)	Wistar rats (N = 30; *in vivo*)	Hypolipidemic activity	Adeneye and Olagunju (2009)
Seed extract	Aqueous extract/400 mg/kg bw (28 days)	Wistar rats (N = 25; *in vivo*)	Hypolipidemic activity	Johnson et al. (2015)
Seed extract	Aqueous extract/200 and 300 mg/kg bw (35 days)	Albino rats fed with high fat diet (N = 30, *in vivo*)	Hypolipidemic activity	Nwangwa and Ekhoye (2013)
Leaf extract	Ethanolic extract/250 and 500 mg/kg/bw (21 days)	Diabetic Wistar rats (N = 40; *in vivo*)	Hypolipidemic activity	Adenowo et al. (2014)
Leaf extract	Aqueous extract/400 mg/kg bw (21 days)	Diabetic rats (N = 36, *in vivo*)	Hypolipidemic activity	Maniyar and Bhixavatimath (2012)
Leaf extract	Aqueous extract/400 mg/kg bw (28 days)	Wistar rats (N = 25, *in vivo*)	Hypolipidemic activity	Johnson et al. (2015)
Leaf extract	Aqueous extract/0.75 and 1.5 g/100 mL (28 days)	Wistar rats (N = 32, *in vivo*)	Hypolipidemic activity	Juarez-Rojop et al. (2014)
Leaf extract	Aqueous extract/200 mg/kg bw (21 days)	Wistar rats (N = 24, *in vivo*)	Hypolipidemic activity	Hasimun et al. (2018)
Leaf extract	Aqueous extract/120 mg/kg bw (28 days)	Albino rats (N = 30, *in vivo*)	Hypolipidemic activity	Ezekwe et al. (2014)
Leaf extract	Aqueous extract/200 mg/kg bw (24 days)	New Zealand rabbits (N = 20, *in vivo*)	Anti-obesity activity	Omonkhua and Onoagbe (2011)
Leaf extract	Aqueous extract/120 mg/kg bw (28 days)	Albino rats (N = 30, *in vivo*)	Anti-obesity activity	Ezekwe et al. (2014)
Leaf extract	Aqueous extract/100 mg/kg bw (30 days)	Hypertense Wistar rats (N = 20, *in vivo*)	Anti-hypertensive activity	Brasil et al. (2014)
Leaf fraction	Ethyl acetate fraction/10–100 μg/mL	RAW 264.7 cells (*in vitro*)	Anti-inflammatory activity	Cao et al. (2024)
Root extract	Aqueous extract/500 mg/kg bw (21 days)	Albino rats (N = 24, *in vivo*)	Hypolipidemic activity	Ezekwe and Chikezie (2017)
Papain	0.125–1 U/mL	Mongrel dog blood (*in vitro*)	Thrombolytic activity	Yang et al. (2023)
Papain	0.1–1 U/mL	Rats with atherosclerosis (N = 15, *in vivo*)	Anti-atherosclerosis activity	Fei et al. (2023)

### 4.1 Extracts

It is known that excessive fat accumulation can be an important contributor to the development of dyslipidemia, hypertension, and alterations conducive to the development of CVDs. Several studies examined the effect of various parts of *C. papaya* in the prophylaxis and treatment of CVDs associated with obesity. For example, the results of Adeneye and Olagunju (Adeneye and Olagunju, 2009) indicate that the aqueous extract from *C. papaya* seeds has hyperlipidemic properties in Wistar rats (100–400 mg/kg administered for 30 days, N = 30). All used doses decreased the levels of total cholesterol, triglycerides, LDL-cholesterol, and increased the concentration of HDL-cholesterol in serum. In addition, the tested extract lowered the atherogenic index. The phytochemical analysis of this plant extract identified the presence of flavonoids, alkaloids, saponins, and tannins. Moreover, the study indicated that the tested extract was safe to use.

Adenowo et al. (2014) noted that the ethanolic extract from papaya leaves (250 and 500 mg/kg/body mass; 21-day supplementation) reduced the levels of triglycerides, total cholesterol, and LDL-cholesterol, together with increased HDL-cholesterol concentrations in diabetic Wistar rats (treated with alloxan (150 mg/kg/body mass; N = 40)). Another study by Maniyar and Bhixavatimath (Maniyar and Bhixavatimath, 2012) also demonstrated the hypolipidemic properties of aqueous extract from papaya leaves (400 mg/kg/body mass; administered for 21 days) in alloxan-induced diabetic rats (N = 36). Phytochemical analysis of the tested extract demonstrated the presence of tannins, alkaloids, flavonoids, saponins, anthraquinones, anthocyanosides, and reducing sugars. More data about the hypolipidemic activity of papaya leaves is also available in the study by Johnson et al. (2015), who administered an aqueous extract of *C. papaya* leaves and aqueous extract of papaya seeds (400 mg/kg/body mass; for 28 days) to Wistar rats. Additionally, other researchers observed the hypolipidemic properties of various extracts from *C. papaya* leaves (such as aqueous extract – 120 and 400 mg/kg; ethanolic extract – 50–300 mg/kg BW) in diabetes-induced animals (Yasmeen and Prabhu, 2012; Sobia et al., 2016; Ukpabi et al., 2019).

Moreover, an aqueous extract from papaya seeds had anti-hyperlipidemic activity in Albino rats fed with a high-fat diet (200 and 300 mg/kg/body mass for 5 weeks; N = 30) (Nwangwa and Ekhoye, 2013).

Hypolipidemic effect was also observed after the administration of aqueous extract from *C. papaya* leaves (0.75 and 1.5 g/100 mL, for 4 weeks) in rats. Moreover, the researchers noted that nitric oxide (NO) metabolites were reduced in these animals. It is known that hyperlipidemia is characterized by the inhibition of endothelial NO synthase (eNOS), and consequently, can result in the formation of various reactive oxygen species (ROS) and impaired endothelium-dependent relaxation. High ROS generation concomitant with lowered effectiveness of antioxidant enzymes can lead to an imbalance between reactive oxygen species formation and the protection against ROS in the organism. Thus, the cardioprotective action observed in this experiment could be linked to the antioxidant activity that the extract had in the tested animals (N = 32) (Juarez-Rojop et al., 2012; Juarez-Rojop et al., 2014). Other results of Juarez-Rojop et al. (2012), Juarez-Rojop et al. (2014) indicate that the bioactive compounds from a chloroform extract of *C. papaya* leaves were steroids. On the other hand, Hasimun et al. (2018) noted that the anti-hyperlipidemic properties of the ethanolic extract from *C. papaya* leaves (200 mg/kg/body mass; for 21 days) is associated with the inhibition of 3-hydroxy-3-methyl-glutaryl-coenzyme A reductase (HMG-CoA reductase, an important enzyme in the synthesis of cholesterol) in Wistar rats with hyper-lipidemia (N = 24).

Moreover, supplementing adult healthy male New Zealand rabbits with an aqueous extract from papaya leaves (200 mg/kg/body mass) for 24 weeks reduced their body weight (Omonkhua and Onoagbe, 2011). Results of Ezekwe et al. (2014), Ezekwe and Chikezie (2017) also showed the hypolipidemic and anti-obesity effects of unripe papaya pulp (120 mg/kg/body mass; for 28 days) in an experimental model of alloxan-induced diabetes in Albino rats (N = 30). Another experiment from the same authors (Ezekwe et al., 2014; Ezekwe and Chikezie, 2017) also demonstrated the hypolipidemic effect of an aqueous extract from papaya root (500 mg/kg/body mass; for 21 days) in Albino rats (N = 24). The major phytocomponents present in the extract were hexadecanoic acid, methyl ester (35.6%) and 10-octadecenoic acid, methyl ester (30.1%), whereas the mi-nor phytocomponents include ergosta-5,22-dien-3-ol acetate (3β,22Ε) (11.5%), dianhydromannitol (7.4%), hexasiloxane-1,1,3,3,5,5,7,7,9,9,11,11-dodecamethyl (7.0%), me-thyl-11-hexadecanoate (4.91%) and octadecanoic acid, methyl ester (3.5%).

Various studies indicate that extracts from different parts of *C. papaya* have anti-hypertensive properties. For example, Brasil et al. (2014) observed this effect by inhibiting the activity of angiotensin-converting enzyme (ACE) *in vitro*. The anti-hypertensive properties of methanolic extract from *C. papaya* leaves (100 mg/kg/body mass; for 30 days) were also noted in spontaneously hypertensive Wistar rats (Brasil et al., 2014). Quercetin, rutin, nicotiflorin, clitorin, and manghaslin were identified in the used extract. Moreover, the crude extract from *C. papaya* fruits (20 mg/kg/body mass) had anti-hypertensive activity in a model of arterial hypertension stimulated by deoxycorticosterone acetate (15 mg/100 g/body mass) in Wistar rats (N = 15) (Eno et al., 2000).

Oxidative stress is also one of the factors conducive to the development and progression of CVDs. Numerous studies (especially *in vitro* models) indicate that extracts from various parts of papaya, especially fruits and leaves, have high contents of antioxidants (such as phenolic compounds and carotenoids) that may reduce oxidative stress (Rani et al., 2023; Asghar et al., 2016; Nugroho et al., 2017; Agada et al., 2021). Jeon et al. (2022) observed that papaya fruits show varying levels of antioxidant activity depending on the part of the fruit and its ripening stage. Regardless of the ripening degree, papaya seeds had a higher concentration of phenolic compounds than the peel-pulp, which corresponded with higher antioxidant potential, especially the 2,2′-azino-bis(3-ethylbenzothiazoline-6-sulfonic acid (ABTS) radical scavenging activity and ferric reducing antioxidant power (FRAP). In addition, NO generation was inhibited to a higher degree in ripe seed extract (200 μg/mL) than in other tested extracts (unripe peel-pulp, unripe seed, and ripe peel-pulp; 200 μg/mL). NO generation inhibition was associated with the suppression of NF-κB activation and iNOS expression in lipopolysaccharide (LPS)-treated RAW-BlueTM cells (*in vitro*).

Rani et al. (2023) noted that an ethanolic extract from *C. papaya* seeds demonstrated significant antioxidant activity, as indicated by its IC_50_ value – 39.4 ± 1.6 μg/mL. The antioxidant potential was measured *in vitro*, with the DPPH assay.

Asghar et al. (2016) studied the antioxidant activity of extracts from papaya leaves using various cultivars, solvents (water, methanol, and ethanol), and maturity stages. Their results demonstrated that the water-extracted mature leaves had the strongest antioxidant properties of any of the other tested types of leaves.

Additionally, various researchers reported that the extracts from *C. papaya* peel have antioxidant potential, which was measured by different methods, including FRAP, ABTS, or DPPH (Salla et al., 2016; Ang et al., 2012; Calvache et al., 2016; Kokila et al., 2016).

Papaya extracts that are rich in papain and other enzymes, and various antioxidants have strong anti-inflammatory properties - they act as suppressors of NF-κB activation (Alhazmi et al., 2021; Cao et al., 2024). Papaya extracts can also regulate inflammatory processes through interference with the mitogen-activated protein kinase (MAPK) signaling pathway (Jeon et al., 2022). Moreover, they can decrease the levels of inflammatory markers, such as tumor necrosis factor-α (TNF-α) and interleukin-6 (IL-6) (Jeon et al., 2022).

In another *in vitro* experiment, (Cao et al., 2024) extracted papaya leaf juice with various organic solvents (hexane, diethyl ether, ethyl acetate, and n-butanol) and not-ed that the ethyl acetate fraction (10–100 μg/mL) had the most outstanding anti-inflammatory properties among the tested extracts. It suppressed the production of NO (IC_50_ = 24.94 ± 2.4 μg/mL) and the expression of pro-inflammatory enzymes (such as iNOS and cyclooxygenase-2 (COX-2), and cytokines (including interleukins IL-1β and −6, and TNF-α) in LPS-induced RAW 264.7 cells. Moreover, the anti-inflammatory mechanisms of the ethyl acetate fraction were associated with the MAPK signaling pathway–the fraction inhibited the phosphorylation of extracellular signal-regulated kinases 1/2 (ERK1/2), c-Jun N-terminal kinases (JNKs), and p38, and prevented the ex-pression of toll-like receptor 4 (TLR4) on the cell surface. The main phytochemicals present in the ethyl acetate fraction analyzed by HPLC-QTOF-MS were flavonoids, especially quercetin and kaempferol glycosides, and caparine. Papaya extracts also demonstrated anti-inflammatory action in animal models as well (Sindhu et al., 2019).

### 4.2 Bioactive properties of papain

There are only few papers about compounds with cardioprotective potential isolated from papaya. Recently, researchers have noted that papain, which is a prominent cysteine protease derived from the latex of *C. papaya*, has cardioprotective activity (Yang et al., 2023; Fei et al., 2023; Jiang et al., 2022). Yang et al. (2023) examined the effect of papain on fibrinogen and other key components of hemostasis using various tests. Authors observed that papain (0.125–1 U/mL) had a dose-dependent blood clot lysis activity - it cleaved fibrinogen chains of Aα, Bβ, and ɤ-bands. It also significantly extended the activated partial thromboplastin time (APTT) and prothrombin time (PT) in an *in vitro* model based on mongrel dog blood. The thrombolytic properties of papain were also examined using a rat tail thrombosis model induced by κ-carrageenan, where the dose of 10 U/kg also showed thrombolytic activity.

Fei et al. (2023) found that papain has anti-atherosclerotic properties not only *in vitro*, but also *in vivo*, in rats with atherosclerosis. This enzyme inhibited CD14 and CD41-induced foam cell formation through the inactivation of MAPK, and phosphoinositide 3-kinase (PI3K)/Akt-NF-κB pathways. Other results indicate that papain has protective effects against monocyte-blood platelet aggregate formation-initiated ex-pression of inflammatory mediators in THP-1 cells (an acute monocyte leukemia cell line) by suppressing NF-κB signaling via the upregulation of miRNA-146a transcription (Lim et al., 2021). More details about the anti-inflammatory potential of papain have been de-scribed by Narayanan (Narayanan, 2025).

**FIGURE 2:**
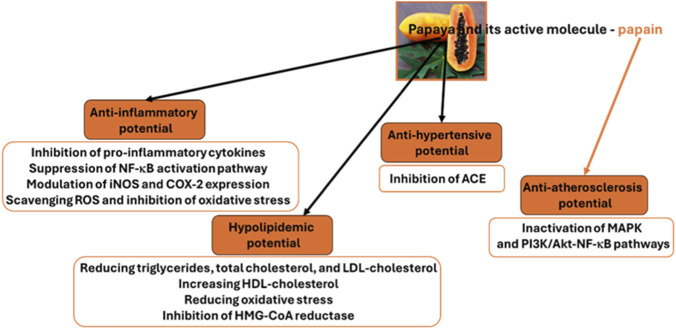
Cardioprotective action of *C. papaya* and its active molecule - papain.

Figure 2 demonstrates the potential molecular mechanisms of cardioprotective activity of *C. papaya* and papain. However, the depicted mechanisms are proposed rather than clinically confirmed.

## 5 Safety of various parts of *C. papaya* and papaya supplements

Several *in vitro*, animal, and human studies have examined the safety of various parts of *C. papaya*. A systematic scoping review by Lim et al. (2021) indicates that the consumption of *C. papaya* leaves in the form of a standardized aqueous extract (20 mg/kg for youngest patients) and in the form of juice (2.5 mL/day for children, and 150 mL/day for adults) is well-tolerated and safe in children (1–12 years) and in adult hu-mans for short durations (<5 days). However, minor gastrointestinal side effects were often observed.

There are concerns about reproductive toxicity and hepatotoxicity in long-term use in animal models. On the other hand, Taychaworaditsakul et al. (2024) observed that 10% ethanolic extract from *C. papaya* leaves (100–5000 mg/kg) exhibits no adverse effect on rats (N = 70) during 180-day oral administration. In this experiment, various physiological and behavioral parameters, including organ and body weights were studied. Results of Ismail et al. (2014) also noted that an extract from *C. papaya* leaves (0.01–2 g/kg bw) did not cause any mortality, abnormalities of behavior, or changes in body weight in rats, when administrated for 13 weeks (N = 10). Moreover, there were no significant differences in hematological markers between treatment and control groups. However, significant differences were observed in biochemical values, such as albumin, creatinine, lactate dehydrogenase (LDH), and total protein, but these changes were not associated with histopathological changes.

Recently, Chaijan et al. (2024) have examined the effect of different extracts from papaya leaves (from three papaya cultivars: Thai Local, Holland, and Khaek Dam) on the viability of RAW 264.7 macrophage cell line activated by LPS. All used extracts (0.98–1000 μg/mL) were non-toxic to RAW264.7 cells. Results of Hyan et al. (2021) also demonstrated that an aqueous extract from *C. papaya* leaves (12.5–400 μg/mL) was not toxic to RAW 264.7 cells after 24 h incubation. Moreover, an ethanolic extract from *C. papaya* leaves had no evident cytotoxicity on T47D cells (Yuliani and Syahdeni, 2020). Recently, Ferguson et al. (2025) have described that *C. papaya* leaf extract is safe in cosmetic formulations.

Rani et al. (2023) examined the toxicity of an ethanolic extract from *C. papaya* seeds (200 mg/kg bw; 14 days) administered orally to Wistar rats. Histopathological examination of the liver and kidney tissues and body weight changes were evaluated. The toxicity studies indicated that the used plant extract was safe for oral administration. Rats exhibited normal behavior, weight stability, and there were no histopathological abnormalities in the liver or kidneys. Other results of Gazwi et al. (2023) also indicate that papaya seed-enriched cakes effectively protect against carbon tetrachloride (CCl_4_)-induced immunotoxicity in rats (N = 48).

Asuquo et al. (2019) noted that the extract from *C. papaya* ripe fruits (500–1500 mg/kg bw) attenuates testicular histomorphological and hormonal changes following alcohol-induced gonad toxicity in male rats (N = 30). In addition, the authors suggest that the protective potential of the used extract is associated with its antioxidant mechanism. More details about the safety aspects of *C. papaya* leaves are demonstrated in other review papers (Krishna et al., 2008; Lim et al., 2021).

The most commonly exploited beneficial properties of supplements from fruits and leaves of *C. papaya* (in the form of juices, capsules, or tablets), are arguably their anti-obesity activity and the effect on the digestive and immunological systems. However, the biological properties of papaya supplements were often demonstrated very poorly. For example, no research that compares the bioavailability of the active compounds in different papaya food products and supplements is available.

## 6 Conclusion

Despite the interest in the biological potential of various parts and preparations (especially extracts) of *C. papaya*, they have not yet been fully explored. Although the extracts from various parts of *C. papaya* (especially from leaves) and their active molecules, such as papain, have been noted to demonstrate multifunctional action on different elements of the cardiovascular system and CVDs, they were studied only in *in vitro* and animal models. In addition, current evidence is largely preclinical and hypothesis-generating. As of yet, there is no concrete clinical evidence for the efficacy or safety of *C. papaya* preparations for the prophylaxis and treatment of CVDs. Therefore, there is a need for more studies, not only on healthy people, but also on people with risk factors for CVDs (for example, high cholesterol, smoking, obesity, or diabetes), who have been supplemented with preparations from various parts of *C. papaya*, especially its fruits and food products.

Comparing the results of different studies can also be difficult, as the outcomes can be different depending on the extraction method, the method used to assess the biological activity, the doses of papaya extracts, and other factors. Therefore, the standardization of extraction methods and biological assays would make comparing different studies easier.

Although the cardioprotective mechanisms of various parts of papaya were re-ported in this mini-review paper to provide a basis for further study and development, more studies are also needed to clarify the cardioprotective mechanisms of papaya bioactive components. There are also still numerous gaps in the understanding of the cardioprotective action of the preparations from various parts of papaya, and a lack of pharmacological trials for the discovery of supplements as a new strategy for pre-venting CVDs.
